# Targeting mitochondrial ClpP: structural insights and therapeutic potential of ClpP agonists in cancer therapy

**DOI:** 10.3389/or.2025.1567860

**Published:** 2025-05-06

**Authors:** Mowei Kong, Yang Yu, Shuai Shao, Chunxiang Zhang

**Affiliations:** Department of Cardiology, The Affiliated Hospital of Southwest Medical University, Southwest Medical University, Luzhou, Sichuan, China

**Keywords:** ClpP protease, mitochondrial dysfunction, targeted cancer therapy, cellular signaling, cancer biology tumor targeting

## Abstract

Mitochondrial “powerhouses” play a central function in cellular metabolism and energy generation. Their dysregulation is directly correlated with a myriad of diseases, among them cancer. The serine protease ClpP, accompanied by its cochaperone ClpX, is a principal homeostatic regulator in mitochondrial function by degrading aberrant proteins in order to preserve mitochondrial integrity. Recently, evidence suggests ClpP is overexpressed in many cancer cells and, as such, is an appealing target for drug therapy. In this review, current information about the structure, physiological function, and therapeutic promise of mitochondrial ClpP in oncology is summarized. We provide an overview about the mechanistic rationale behind ClpP agonists as novel anticancer drugs, their regulation in cell signal transduction, and the major challenge in the creation of small molecules that specifically activate human ClpP, but not bacterial ClpP. The review highlights the therapeutic promise of ClpP agonists as a novel approach in cancer therapy, presenting their prospective potential for cancer treatment by focusing on an unexplored mitochondrial target.

## 1 Introduction

Mitochondria are the cell powerhouses, capable of producing ATP through oxidative phosphorylation in order to provide energy for cellular function. Mitochondrial oxidative phosphorylation is primarily catalyzed by the electron transport chain (ETC), which consists of four multi-subunit enzyme complexes (Complex I-IV). These complexes transfer electrons derived from the tricarboxylic acid cycle, generating a proton gradient that drives ATP synthesis (1). Mitochondria are semi-autonomous and functionally compartmentalized organelles. They encode 13 proteins within their own genome, while over 1,000 other mitochondrial proteins are encoded by the nuclear genome, translated in the cytosol, and subsequently targeted to various mitochondrial compartments (2, 3). Mitochondria possess their translation apparatus and degradation machinery, among which is highly conserved in humans, the caseinolytic protease P (ClpP), a mitochondrial matrix serine protease. In normal situations, ClpP exercises its hydrolytic activity in a regulation-dependent way by the molecular chaperone ClpX. In oxidative stress in cells, particularly in situations with accumulated unfolded protein, ClpP plays a critical hydrolytic function, regulating mitochondrial protein quality and mitochondrial proteome homeostasis (3, 4). ClpP agonists can hydrolyze substrate protein such as respiratory chain-related protein regardless of ClpX, which is reported as a novel tumor treatment method (5). ClpP is capable of hydrolyzing damaged and misfolded protein, thereby maintaining mitochondrial proteome homeostasis and integrity of mitochondrial function (4). In physiologic situations, ClpP hydrolytic activity is ClpX-dependent. The AAA+ ATPase HsClpX possesses the function of forming a hexameric architecture, with which the tetradecameric ClpP forms a complete ClpXP complex with hydrolytic activity (6, 7). ClpX recognizes, binds, and unfolds substrates in ATP-dependent manner and then translocates them to barrel-shaped hydrolytic chamber HsClpP for degradation. In the last few decades, with more and more in-depth studies about biological function and biological function in human diseases in HsClpP, more and more evidence reveals that defects in ClpP correlate with the occurrence and progression of numerous kinds of diseases like tumors, neurological diseases, and metabolic diseases. A number of tumor-related reports reveal that ClpP is involved in cancer promotion in various cancers like acute myeloid leukemia, breast cancer, pancreatic cancer, and colon cancer (5, 6). However, there were no reports about anti-tumor biological mechanisms and signal pathways of ClpP agonists till now. The novelty of this review is that it brings novel findings and potential anti-tumor drug targets for ClpP, so we can obtain safer and more effective anti-cancer drugs.

## 2 Molecular architecture and mechanism of ClpP protease

**FIGURE 1:**
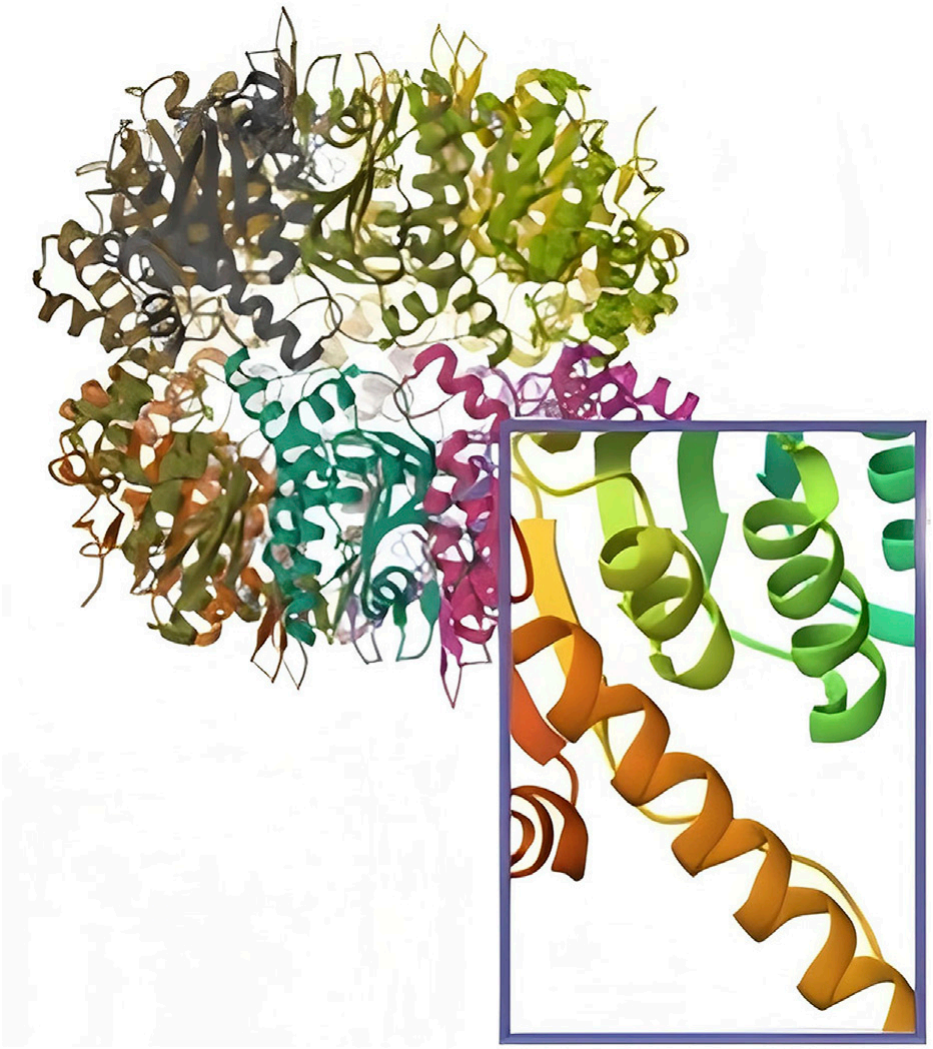
The structure of ClpP and in human mitochondria, ClpP exists as a low-activity heptamer under physiological conditions.

ClpP is a serine protease located in the mitochondrial matrix that exhibits its hydrolytic activity through a catalytic triad of S153-H178-D227 (7). ClpP was initially identified as a gene in *Escherichia coli*, and the ClpP amino acid sequence is highly conserved in the majority of organisms (8). In bacteria, ClpP exists as a homotetradecamer, while in human mitochondria, ClpP exists as a low-activity heptamer under physiological conditions (Figure 1).

In human mitochondria, ClpP consists of 277 amino acid residues, where the N-terminal 56 amino acids are the mitochondrial targeting sequence (MTS), and the central protease domain in combination with ClpX performs its hydrolytic activity (9). ClpX consists of 633 amino acid residues, including the MTS, the Zinc-binding domain (ZBD), and the AAA+ (ATPases associated with various cellular activities) ATPase domain (9). Both ClpX and ClpP are nuclear-encoded genes, which are translated in the cytosol and directed to the mitochondrial matrix by the MTS sequence (10). The ZBD domain of ClpX can recognize and bind the substrate proteins, while the AAA+ ATPase domain energizes through ATP hydrolysis (10).

**FIGURE 2:**
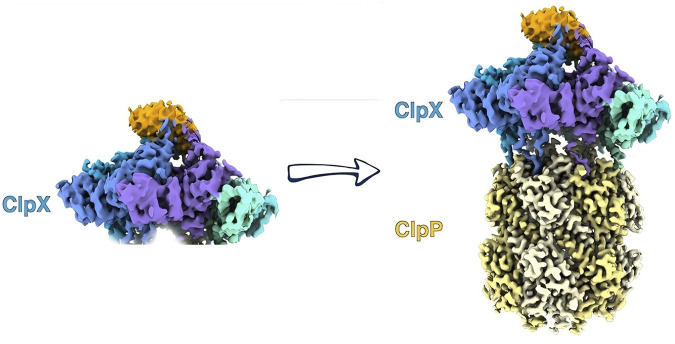
ClpX binding to ClpP triggers a conformational change, shifting ClpP from an inactive heptamer to an active tetradecamer state.

Although the structure of the human ClpXP complex has not been determined, the binding mode of human ClpXP can be inferred from the structures of ClpXP complexes from diverse bacterial sources. ClpX binding to ClpP induces the shift of ClpP from an inactive heptamer conformation to an active ten-tetramer conformation (Figure 2). ClpX exists as a monomer to hexamer complex under physiological conditions but in the presence of ATP-yS can be a stable hexamer (11). On binding to ClpP, two hexamers of ClpX bind to each end of the ClpP tetradecamer. The loop region between L439-G440F441 and E436-G450 of ClpX binds to the N-terminal hydrophobic chamber of ClpP and modulates the substrate hydrolytic activity of ClpP (12). ClpX, as a molecular chaperone, can recognize the unfolded peptide sequence of the substrate, using the energy from ATP hydrolysis to unfold and extend the substrate and transport it into the active chamber of ClpP (12). Subsequently, ClpP hydrolyzes the substrate into oligopeptides containing just 7 to 8 amino acid residues and releases the hydrolyzed substrate through the axial pore in the center of the ClpP chamber.

## 3 ClpP’s role in mitochondrial homeostasis and tumor development

The mitochondrial unfolded protein response (UPRmt) is a response that keeps mitochondria’s proteins under control.

It is activated when cells are distressed, using special proteins to correct misfolded proteins and cause new ones to fold correctly (13). The primary physiological functions of ClpP include participation in mitochondrial quality control, involvement in the UPRmt, and an effect on tumor growth and development (13). It is said that ClpXP in mitochondria can selectively degrade the subunits of the defective respiratory chain complexes I and II, thus saving biological energy as the respiratory chain complexes do not need to be rebuilt (14). Jae et al. found that knockdown of ClpP led to an increase in protein levels of misfolded succinate dehydrogenase B (SDHB) and a loss of function of respiratory complex II (RCII) that disrupted oxidative phosphorylation (OXPHOS) and led to ATP synthesis being compromised and increased cellular oxidative stress levels (15). Moreover, ClpXP is able to regulate proteins that are involved in mitochondrial RNA stability and thereby affect mitochondrial gene expression, and through ERAL1 protein amount regulation, control the level of mitochondrial protein synthesis (16). Therefore, ClpXP maintains regular mitochondrial function and energy metabolism by substrate protein level regulation. The major mitochondrial proteases include ATP-dependent proteases like ClpP and LonP1, which have the potential to degrade damaged proteins (17).

When, under various stress conditions, there is a huge accumulation of unfolded, misfolded, and broken-down proteins in the mitochondrial matrix, the UPRmt promotes the synthesis of mitochondrial molecular chaperone proteins and proteases, thereby aiding the restoration of mitochondrial protein homeostasis (18, 19). Rapid cancer cell growth and proliferation often result in hypoxic conditions, mtDNA mutations, and ROS production, causing mitochondrial dysfunction and accumulation of unfolded and misfolded proteins (19). Experiments have proved that UPRmt activation ensures cancer cell survival and evasion of apoptosis, and UPRmt is activated in breast cancer tissues alone but not in normal breast tissues (20, 21). Activated UPRmt also increases the expression of proteases such as ClpP, degrading misfolded or damaged proteins, alleviating environmental stress, and promoting the survival of tumor cells (22). Immunohistochemical staining of clinical specimens indicated that ClpP is expressed more highly in cancer cells than in para-cancerous tissues (23).

In solid tumor tissues and organs of the lung, stomach, liver, thyroid, bladder, breast, ovary, prostate, testis, and central nervous system, the protein expression levels of ClpP have been shown to be remarkably upregulated (24, 25).

Similarly, in acute myeloid leukemia (AML), upregulation of ClpP expression is observed (26). Experiments have found that in PC-3 tumor cell lines of human prostate cancer, downregulation of ClpP expression leads to the inhibition of growth and proliferation of tumor cells and a dramatic reduction in cloning ability (27, 28). In MCF-7 tumor cell lines of human breast cancer, however, the proliferation of tumor cells is not affected by the downregulation of ClpP expression [29]. In human pancreatic cancer BxPC-3 tumor cell lines, by creating a doxycycline-induced ClpP expression system, compared with the wild-type cells, the doxycycline-induced group had inhibited tumor cell growth and proliferation (30). In lung squamous cell carcinoma LUSC tumor cells, by creating a doxycycline-overexpressing Y118A ClpP mutant protein, in a xenograft tumor model of mice, compared with the wild-type group, mouse tumor volume reduction was observed with doxycycline intake (31). Briefly, ClpP regulates the growth of most tumor cells, and a correlation between tumor size and levels of ClpP expression has been observed in mouse models, making it a potential target for tumor therapy.

## 4 Advances in ClpP agonist discovery and their anti-tumor activities

Recent research has shown that ClpP hyperactivation can degrade substrates like respiratory chain complex I and II, tricarboxylic acid cycle-related enzymes, and mitochondrial translation-related proteins, thereby disrupting cellular mitochondrial oxidative phosphorylation, mitochondrial protein synthesis, and energy production, which are basic life processes (32, 33).

Different tumor cells rely heavily on the oxidative phosphorylation pathway for their energy needs. ClpP agonists exhibit antitumor activity by inducing the degradation of respiratory chain complexes, thereby disrupting the oxidative phosphorylation pathway and blocking energy supply (32). Based on the chemical structure of agonists, the exemplary representatives of ClpP agonists are divided into five categories: ADEP (acyldepsipeptide) class, the D9 class of heterocyclic arylamines, the Imipridone class represented by ONC201, ZG111 class, and ZK53 class (34). ADEP was initially isolated from the fermentation broth of *Streptomyces* hawaiiensis for antibacterial application.

After genomic screening of ADEP-resistant genes, ClpP was found to be a potential target of ADEP (35). Wong et al found that ADEP and its analogues can also bind to the hydrophobic pockets at the interface of two molecules of ClpP, disrupting the binding site and activity of ClpX, and activating ClpP (36). *In vitro* enzymatic assays found that ADEP-28 and ADEP-41 have apparent dissociation constants Kd for substrate FITC-Casein of 120 nmol/L (37). ADEP-41 activates ClpP to initiate the intrinsic cellular apoptosis signaling pathway, thereby disrupting the mitochondrial morphology and function of human cervical cancer HeLa cells, undifferentiated human neuroblastoma SH-SY5Y cells, and human osteosarcoma U2OS cells, and causing tumor cell apoptosis (38). Structural biology methods revealed that ADEP-28 causes ClpP to change from a heptamer state to a compact tetradecamer state (37). The pore diameter of ClpP-ADEP28 is wider and the axial length is longer than in the Apo state, making it easier for the loop region of substrate proteins to pass through the hydrolytic chamber of ClpP and be degraded (39). Therefore, ADEP class compounds can bind and make ClpP change from a low-activity heptamer form to a high-activity tetradecamer form with more hydrolytic activity. Stahl et al. used high-throughput screening technology combined with fluorescence substrate proteolysis analysis methods to screen heterocyclic arylamines that selectively activate human ClpP with low selectivity for bacterial ClpP, represented by D9 (40).

D9 displays concentration-dependent activity against ClpP protease activity and also can activate the mutant Y118A ClpP (40). Further, in human breast cancer SUM159 cells, D9 activates ClpP, promoting the degradation of mitochondrial transcription factor A (TFAM) and mitochondrial Tu translation elongation factor (TuFM) (41). The most explored ClpP agonists are the Imipridone class exemplified by ONC201, which has a unique tricyclic ring skeleton. ONC201 was initially discovered as a non-p53 dependent inducer of the transcription of the tumor necrosis factor-related apoptosis-inducing ligand (TRAIL) gene. The target of ONC201 was initially unknown, and it was believed that ONC201 possessed the capability to antagonize dopamine receptors (DR) D2/D3 (42, 43). However, the antitumor activity of ONC201 could not be fully accounted for by its dopamine receptor antagonism, and compounds with more potent dopamine receptor D2/D3 antagonism did not exhibit more potent tumor activity relative to ONC201 (44). ONC201, with the advantages of antitumor activity, high safety, high blood-brain barrier permeability, high oral bioavailability, and stable pharmacokinetic profile, has been applied in clinical trials.

Studies have shown that ONC201 not only exhibits therapeutic activity against solid tumors but also impacts the growth and behavior of tumor stem cells, tumor-associated fibroblasts, and immune cells within the tumor microenvironment (42). Recent research shows that ONC201 triggers the activation of ClpP to degrade a cascade of mitochondrial proteins, including respiratory chain complexes, to impair mitochondrial protein homeostasis, devastate normal mitochondrial function, and trigger a cascade of pro-apoptotic signaling pathways to exert antitumor activities (44). In triple-negative breast cancer cell lines, MDA-MB231 and SUM159 tumor cells treated with ONC201 show a significant increase in the expression levels of C/EBP homologous protein (CHOP) and activating transcription factor 4 (ATF4), markers of endoplasmic reticulum stress and integrated stress response (ISR), respectively (45). At the same time, the phosphorylation of ERK and AKT is increased, and the protein level of TFAM and TuFM decreases, triggering a cascade of signaling pathway and ISR, and the growth and proliferation of tumor cells are inhibited and tumor cell apoptosis is induced, ultimately achieving the goal of antitumor activity (45). After ONC201 activates ClpP, it leads to the degradation of other mitochondrial proteins, including proteins of the respiratory chain and proteins necessary for mitochondrial translation, thereby inhibiting processes such as mitochondrial oxidative phosphorylation, mitochondrial translation, and lipid metabolism (42, 44). Additionally, cell morphology observations reveal mitochondrial ridge breakdown and increased ROS levels. Biological experiments have shown that ClpP activation by ONC201 can suppress the expression of the oncogene C-MYC, inhibit the mTORC1 signaling pathway, upregulate the expression of death receptor 5 (DR5), and activate caspase-8. These effects ultimately lead to tumor cell apoptosis and inhibition of tumor growth (5). As a monotherapy and in combination with other drugs, ONC201 showed promising antitumor activity and outstanding safety in phase I and phase II clinical trials in patients with gliomas of the brain, adrenal tumors, and serous endometrial cancer (46). ONC201 is now approved to proceed to phase III clinical trials for the treatment of H3K27M-mutant gliomas. Based on the parent nucleus of ONC201, through structural modification, a compound series like TR57 and TR107 is synthesized (41). Among them, TR57 inhibits the growth and proliferation of SUM159 tumor cells of breast cancer (41) by inducing ISR and endoplasmic reticulum stress response marker ATF4 and CHOP protein expression levels, inhibiting TFAM and TuFM protein expression levels, and inhibiting colony formation ability (41).

TR107 exhibits nanomolar antitumor activity against triple-negative breast cancer SUM159 and MDA-MB231 cells and degrades the essential proteins of oxidative phosphorylation and tricarboxylic acid cycle pathways via ClpP by generating a ClpP-KO cell line (47). Geng et al. designed and synthesized a compound series represented by 16z on the basis of ONC201’s chemical structure through the modification of the position of the substituent groups (48). Upon testing the lead compounds on a number of tumor cell lines, the lead compound was found to be most sensitive to colorectal cancer HCT116 cells.

Through structure optimization, 16z compound was attained, and cell-based assays showed that 16z is a 40 nmol/L IC50 compound against colorectal cancer HCT116 cells and significantly reduced the protein levels of respiratory chain complex, dissipated mitochondrial membrane potential, triggered ROS release, activated ISR, inhibited colony formation, induced cell cycle arrest, and thus induced cell apoptosis (48). By contrast, Zhou et al. discovered a new type of ClpP agonist, ZK53, in 2023. Different from the above multi-ring rigid structures, ZK53 is based on a more flexible six-membered ring structure, with two termini connected to benzene rings with varied substituents (24). In selectivity, ZK53 can activate human ClpP alone and does not bind or activate *E. coli* EcClpP and *Staphylococcus aureus* SaClpP, thus not suppressing the growth of intestinal flora. By genetic and omics analysis, it was discovered that ZK53 is able to activate ClpP, induce degradation of mitochondrial respiratory chain complex, disrupt mitochondrial membrane potential levels, induce ROS release, influence mtDNA copy number, inhibit oxidative phosphorylation processes, disrupt the morphology of mitochondrial ridges, and finally cause mitochondrial dysfunction, thus inducing cell apoptosis. ZK53 inhibits the growth and proliferation of human lung squamous cell carcinoma H1703, H520, SK-MES-1, and H226 tumor cells (24). Further mining of transcriptome data found that ZK53 also has effects on cell cycle signaling pathway regulation, and further found that ZK53 downregulates the gene and protein levels of cyclin D1, cyclin E2, and cyclin-dependent kinases (CDKs), which results in tumor cell arrest at the G0/G1 phase, thereby inhibiting cell growth and proliferation to play antitumor roles (24).

## 5 Summary and prospects

ClpP, as one of the serine proteases that participate in mitochondrial protein homeostasis, takes part in the UPRmt and maintains mitochondrial protein homeostasis. Recent studies have also explored the role of ClpP in cancer treatment. For instance, Wedam et al. (49) investigated the potential of ClpP agonists in breast cancer, highlighting their ability to induce mitochondrial dysfunction and apoptosis. Similarly, Nouri et al. (50) reviewed the biological functions of ClpP and its emerging role as a cancer therapeutic target. Cormio et al. (51) proposed ClpP as a possible prognostic marker and therapeutic target in various cancers. While these studies provide valuable insights, our review aims to offer a comprehensive overview of ClpP agonists, focusing on their structural insights, physiological functions, and therapeutic potentials across multiple cancer types. Our work uniquely integrates the latest findings on ClpP agonists’ mechanisms and their potential as novel anticancer agents.

In recent years, it has been confirmed that ClpP can be used as an antitumor target. Among which, ClpP agonists replace the function of ClpX, bind to and activate ClpP to form a tetradecameric barrel structure, and trigger cell apoptosis signaling pathways by degrading proteins of the respiratory chain complex, causing ROS release, impairing mitochondrial function, and activating ISR, ultimately leading to tumor cell death. Table 1 lists the representative ClpP agonists, their activities against tumor cells, pharmacological effects, among which Imipridone-class compounds represented by ONC201 have advanced to phase III clinical trials and demonstrated excellent antitumor effects. However, due to the conservation of bacterial and human ClpP structures, side effects such as intestinal flora dysbiosis could appear when applying antitumor medications. By carrying out in-depth research on the ClpP target, novel small molecules that selectively bind to and activate human ClpP have also been identified. In the meantime, by conducting in-depth research on antitumor biological mechanisms, the signaling pathways and biological mechanisms triggered by small molecule activation of ClpP have also been clarified, thus promoting the research on ClpP agonists.

**TABLE 1:** Overview of ClpP agonists, their effects and mechanisms.

Agonist name	Pharmacological effects	Mechanism of action	Tumor cell types	Chemical structure features	Reference numbers
ADEP	Antibacterial, Antitumor	Replaces ClpX function, activates ClpP	Various tumor cells including HeLa, SH-SY5Y, U2OS	Lipopeptide extracted from *Streptomyces* hawaiiensis	(36, 37, 38)
D9	Antitumor	Enhances ClpP proteolytic activity	SUM159, Various tumor cells	Heterocyclic arylamines	(39, 41)
ONC201	Antitumor	Degrades mitochondrial proteins, promotes apoptosis	Various solid tumors	Unique tricyclic ring skeleton	(42, 43, 44)
TR57	Antitumor	Promotes ISR and ER stress	Breast cancer SUM159	Derived from ONC201 structure modification	(41)
TR107	Antitumor	Degrades key proteins of oxidative phosphorylation	Triple-negative breast cancer SUM159 and MDA-MB231	Derived from ONC201 structure modification	(47)
16z	Antitumor	Destroys mitochondrial function	Colorectal cancer HCT116	Changed substituent group positions based on ONC201	(48)
ZK53	Antitumor	Destroys mitochondrial function, promotes cell cycle arrest	Lung squamous cell carcinoma H1703, H520, SK-MES-1, H226	Flexible six-membered ring structure with different substituents on benzene rings	(24)
